# Speed-of-sound imaging using diverging waves

**DOI:** 10.1007/s11548-021-02426-w

**Published:** 2021-06-23

**Authors:** Richard Rau, Dieter Schweizer, Valery Vishnevskiy, Orcun Goksel

**Affiliations:** Computer-assisted Applications in Medicine group, ETH Zurich, Zurich, Switzerland

**Keywords:** Speed-of-sound imaging, Computer tomography, Reconstruction, Inverse problem, Aberration correction

## Abstract

**Purpose.:**

Due to its safe, low-cost, portable, and real-time nature, ultrasound is a prominent imaging method in computer-assisted interventions. However, typical B-mode ultrasound images have limited contrast and tissue differentiation capability for several clinical applications.

**Methods.:**

Recent introduction of imaging speed-of-sound (SoS) in soft tissues using conventional ultrasound systems and transducers has great potential in clinical translation providing additional imaging contrast, e.g., in intervention planning, navigation, and guidance applications. However, current pulse-echo SoS imaging methods relying on plane wave (PW) sequences are highly prone to aberration effects, therefore suboptimal in image quality. In this paper we propose using diverging waves (DW) for SoS imaging and study this comparatively to PW.

**Results.:**

We demonstrate wavefront aberration and its effects on the key step of displacement tracking in the SoS reconstruction pipeline, comparatively between PW and DW on a synthetic example. We then present the parameterization sensitivity of both approaches on a set of simulated phantoms. Analyzing SoS imaging performance comparatively indicates that using DW instead of PW, the reconstruction accuracy improves by over 20% in root-mean-square-error (RMSE) and by 42% in contrast-to-noise ratio (CNR). We then demonstrate SoS reconstructions with actual US acquisitions of a breast phantom. With our proposed DW, CNR for a high contrast tumor-representative inclusion is improved by 42%, while for a low contrast cyst-representative inclusion a 2.8-fold improvement is achieved.

**Conclusion.:**

SoS imaging, so far only studied using a plane wave transmission scheme, can be made more reliable and accurate using DW. The high imaging contrast of DW-based SoS imaging will thus facilitate the clinical translation of the method and utilization in computer-assisted interventions such as ultrasound-guided biopsies, where B-Mode contrast is often to low to detect potential lesions.

## Introduction

Ultrasound (US) imaging is indispensable in computer-assisted interventions from surgical navigation to operative guidance, thanks to its being a low cost, non-ionizing, portable, and real-time imaging modality. Typically, US is known as a B-Mode imaging method that maps echo amplitudes indicating local tissue reflectivity. Nevertheless, B-mode images do not necessarily provide sufficient contrast for certain anatomical structures and pathological conditions. Elastography, for example, creates images of local tissue elasticity in terms of shear modulus which may indicate pathological state [35]. Speed-of-sound (SoS) and acoustic attenuation are other tissue parameters that are known to have valuable differentiation capability [2]. To characterize and map these acoustic properties, transmission-based computed tomography (CT) uses specialized US imaging setups for arrival time and power-loss computation with water-bath suspension of the breast anatomy[21, 22]. Such transmission-mode US imaging systems do not rely on echoes, i.e. US reflections at tissue interfaces but rather record a transmitted signal directly with another transducer at an opposite location, e.g. in a ring structure. It was shown that with such setups quantitative assessment of SoS bears tremendous potential for breast cancer detection [19–21]. In comparison to shear-wave elastography, SoS was found to lead to a better *ex vivo* tissue differentiation [8] with high specificity for benign and malignant tumors [10, 11, 18]. However, in transmission-mode US imaging, a double-sided access and hence a water-bath suspension of the anatomical structure is required, e.g., with two opposing [20], ring shaped [5] or full 3D [7] transducer geometries. These require costly and non-portable systems and an additional technician to operate, with a limited application on only submersible body parts, e.g. the breast and the extremities.

Novel US contrast modalities as above and their tissue differentiation capability are highly relevant also for image-guided planning and navigation. For instance, for US-guided needle biopsies as for the breast, prostate, and liver lesions, the visibility of potentially suspected regions in the US images could allow to target those specific locations; in a real-time fashion potentially enabling sensitivity for lesions otherwise invisible in B-mode. Nevertheless, dedicated and bulky transmission imaging setups mentioned above preclude interventional applications of these novel contrast modalities, due to limitations to submersible anatomy. Even for the breast, carrying out interventions, such as biopsies, in a water bath and within the limited space of a bulky setup would be infeasible in the current clinical realm. Reliable imaging of such modalities with conventional clinical hand-held US transducers is an essential step in enabling their interventional applications.

For hand-held imaging, the use of a passive reflector as an acoustic mirror and timing reference was proposed in [28, 32] for SoS reconstruction from time-of-flight measurements to the reflector placed at a known distance from the transducer. This was later extended to imaging acoustic attenuation [25] and its spectral mapping [26] by referencing measurements to water-bath calibration of the reflector appearance. Obviating the need for a reflector, small misalignments between images acquired at different plane-wave (PW) angles were used in [14] to reconstruct SoS distribution using a Fourier domain reconstruction approach. In [31], SoS reconstruction in the spatial domain was shown to yield improved accuracy and less artifacts. In [34] it was proposed for PW transmits to adapt receive apertures dynamically when beamforming different image locations to minimize spatial point-spread function (PSF) variation, in order to improve displacement estimation used for SoS3 reconstruction. Deep-learning-based variational neural network approaches for inverse-problem of SoS have been demonstrated to yield fast and robust reconstructions in [3, 39, 40].

In clinical settings, several works have studied SoS imaging using transmission-mode and water-submerged systems, e.g. for breast tissue classification [17], solid mass differentiation [13], and imaging human-knee [41]. Using conventional transducers in pulse-echo mode, SoS has been studied clinically for quantifying muscle loss [30] and breast density [29], as well as for differential diagnosis of breast cancer [27]. SoS maps can also help to correct for beamforming delays and hence to improve any other US imaging modality. Typical beamforming assuming a constant SoS computes incorrect delays, not only reducing B-mode image resolution but potentially also affecting any following image processing such as texture analysis, tumor classification, segmentation, image translation, and displacement estimation for elastography. With the knowledge of SoS distribution, such aberrations can be corrected as demonstrated in [1, 15, 24].

Despite promising studies, robust pulse-echo SoS reconstructions using conventional transducers are still challenging. Compared to differential diagnosis, where the real-time aspect is less of an essence and presegmented regions may potentially be used as priors [12], e.g. for quantification of region averages, interventional imaging and image-guided applications with real-time probe manipulation depend on robust image reconstructions without priors. State-of-the-art SoS techniques using PW transmit sequences are shown herein to yield subobtimal imaging due to PSF distortions and consequent displacement measurement errors. To address this, we herein propose a transmit sequence with diverging waves (DW) to minimize aberration artifacts and thus yield improved reconstructions. Although PW sequences are known to allow for high frame-rate and high quality images [23, 36], DW (also termed synthetic transmit aperture imaging) benefit from lower aberration effects and have been presented over the recent decades for several other ultrasound imaging modalities, including B-Mode, Doppler, and Vector Flow imaging [16, 36]. We herein study the feasibility of utilizing DW for SoS imaging, comparatively to PW, also considering the effect of PSF centering via adapted receive apertures.

## Motivation

**Fig. 1:**
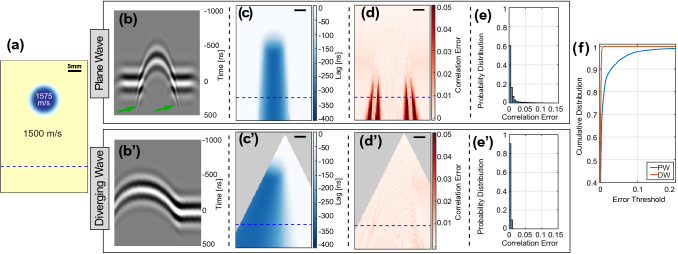
Wavefront aberration comparison in simulations using acoustic recordings in the entire imaging field. (**a**) Heterogeneous SoS distribution with a circular Gaussian-smoothed inclusion of 1575 m/s on a 1500 m/s substrate. The wavefronts arriving at the dashed line in (**a**) are shown (**b**) for $0^\circ$ PW transmit and (**b’**) for a DW. The time-of-arrival (ToF) on the y-axis in (**b**,**b’**) is referenced to the ToF of wavefront peak for a homogeneous simulation without any SoS inclusions. Green arrows indicate columns of aberration effects due to diffraction. Having placed virtual receivers across entire FoV and cross correlating arriving signals with signals from the homogeneous setting, local (**c/c’**) lags and (**d/d’**) correlation errors ($=$ $1-$correlation coefficient) are shown. Note that, for the single DW case, given the directivity of finite-width transducer elements, the acoustic energy is delivered within a triangular opening (shown as masked in **c’** & **d’**), outside of which beamforming and hence time-lag computations are infeasible with sufficient SNR, and are thus also omitted from SoS reconstructions. (**e/e’**) Probability distribution of the correlation error based on three {-10,0,10}$^\circ$ PW and 32 Tx-element DW datasets. (**f**) Cumulative distribution function from (**e/e’**), illustrating the superiority of DW

To demonstrate the effects of a wavefront choice on aberration related artifacts and to motivate the use of diverging waves in this context, we first present a simulated example below (cf. Fig. 1) comparing PW and DW. Using the MATLAB toolbox k-Wave [37], we simulate different transmit schemes and record the spatio-temporal acoustic signal at each and every point in the entire imaging field-of-view (FOV). For both transmit settings, we run two simulations: one with an SoS inclusion and one for a homogeneous case with no SoS inclusions, in order to comparatively quantify the effect of aberrations introduced by the inclusion. Consider the wavefronts arriving at a certain depth (e.g., marked with the dashed line in Fig.1a). As expected, the wavefronts passing through the inclusion would arrive earlier at such depth, compared to a no-inclusion scenario, cf. Fig. 1b/b’. Besides such earlier arrival, one can observe the strong aberration effects below the edges of the inclusion for the PW case (shown with the arrows in Fig. 1b), mainly caused by diffraction. Such aberrations could aggravate when the echos are considered, and would largely hinder any post-processing such as delay estimation for SoS reconstruction. To demonstrate this, we perform here a delay estimation only for the transmit side using normalized cross-correlation (NCC) between spatio-temporal data at all image points for with-inclusion and no-inclusion cases. Fig. 1c/c’ show the estimated delays based on cross-correlation lags and Fig. 1d/d’ show correlation errors (i.e. $1-$CorrelationCoefficient) in delay estimation, resulting from PW and DW, respectively. As seen in Fig. 1d, delay estimation accuracy is quite low with PW transmits, compared to DW. This can be more generally stated via statistics from multiple PW and DW settings, shown in Fig.1e/e’ as probability density functions, where the PW case is seen to have errors further away from zero. To better illustrate this, the cumulative distribution functions are plotted in Fig.1f, which indicates the number of highly aberrated readings that forestall accurate displacement estimation. As can be seen, given any NCC tolerance/threshold, DW would yield much superior displacement estimation than PW, for aberrations typical to expect in in-vivo tissue. For instance, for an NCC tolerance of minimum 0.99, 20% of PW readings would be below this threshold, while all DW readings would be within bounds – which is a large margin considering the single small inclusion given a large homogeneous FOV.

In the context of SoS imaging, such wavefront distortions lead to incoherent signal summations in receive beamforming, thus potentially corrupting displacement estimations, which in turn degrade SoS reconstruction. By reducing wavefront aberrations, DW can yield improved SoS imaging, as shown later in our experiments.

## Methods

**Fig. 2:**
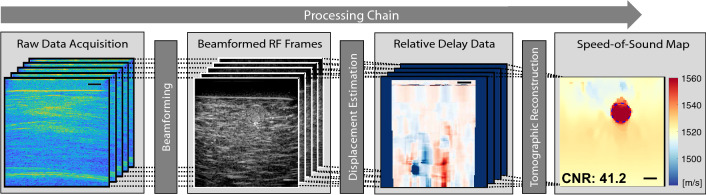
SoS imaging Processing chain: Raw channel data acquired with different Tx sequences is beamformed and apparent displacements are computed. Based on the respective Tx-Rx wave paths, the forward problem of relative delays is formulated as a linear system and tomographically reconstructed

We herein use a limited-angle computed tomography (LA-CT) reconstruction method in the spatial domain, similarly to [31] with the adaptations described below. The fundamental imaging principle and an overview of the data processing is sketched out in Fig. 2. First, raw data is acquired based on a PW or DW sequence, both of which involve multiple transmits (Tx) and after each Tx a receive (Rx) recording of RF data on all element channels. For a DW Tx, a single element emits a narrow band-limited pulse. For a PW Tx, all elements emit such a pulse, with a fixed time-delay between neighbouring elements to angulate the wave-front, where necessary. Rx recordings from a set of multiple such transmissions (Tx) is the input herein for the reconstruction of an SoS frame. Then, separately for each Tx, these Rx signals are beamformed into spatial RF frames, between which apparent local displacements are estimated to be next used to reconstruct a SoS map.

*Beamforming.* To beamform with the received raw channel data from the PW or DW transmit sequences (cf. Fig. 2), we herein employ a conventional delay-and-sum algorithm. Delays are computed with an assumed constant SoS of $1500\,\mathrm{m}/\mathrm{s}$ for the simulations and $1470\mathrm{m}/\mathrm{s}$ for the phantom data acquisition. For both transmit schemes and all RF frames, beamforming is performed on a fixed Cartesian grid aligned with the transducer surface, for a fixed sampling space for the subsequent displacement estimation between these frames.

**Fig. 3:**
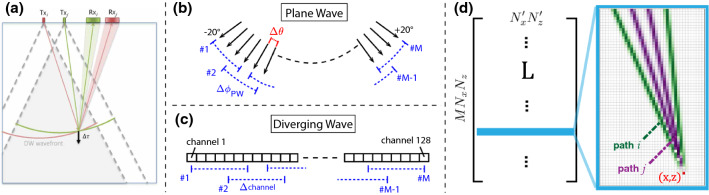
**a** Tx and Rx paths for two DWs *i*, *j* (adapted RX aperture). **b** PW (arrows indicating PW normals) with displacement tracking between $\varDelta \theta$. Relative delay data obtained by accumulating increments to an angular disparity $\varDelta \phi _{PW}$. **c** DWs are created with a single channel. **d** A sample row of the differential path matrix **L** linking SoS distribution and relative delay data at pixel (*x*, *z*) (positive/negative values for path *i*/*j*

We present results with two different beamforming choices: with *full* Rx aperture and with an *adapted* Rx aperture. In full Rx aperture case signals from all channels are fed into beamforming, while still subjected to dynamic aperture per imaging depth (F-$\hbox {number}=1$), which results in Rx aperture staying centered above each beamformed image point. As Tx arrival directions to a point keep changing with each Tx, this yields a PSF varying between different transmits, impeding the subsequent displacement estimation. This is remedied with an adapted Rx aperture (Fig. 3a), centered for each beamformed image point such that the PSF between Tx events to be displacement estimated is aligned as described in [34]. Depending on the RX aperture, PSFs can be aligned at different angles for the same Tx event. Each Tx event here is beamformed with $N_{\mathrm{psf}}=3$ PSF angle alignments: $\psi _{\mathrm{psf}}=0^\circ$ and $\pm 15^\circ$, similar to the settings in [24]. For all transmits, we utilize a fixed Cartesian beamforming grid of $N_x\times N_z$.

For the apparent displacement estimation between beamformed RF frames (cf. Fig. 2), we use a normalized cross-correlation algorithm in the axial direction, similarly to [31]. Depending on the alignment of PSF in beamforming, the estimated displacements are then corrected in the corresponding Tx-Rx direction, i.e. by multiplying them by $\cos (\psi _{\mathrm{psf}})$. For a constant computational complexity and to keep the data input into the reconstruction constant, we herein compute the relative delay data for a fixed number of $M=9$ combinations, yielding an apparent displacement vector of $\varvec{\varDelta \tau } \in {\mathbb {R}}^{M N_x N_z}$. Note that for the adapted Rx aperture case, each Tx sequence is beamformed with $N_{\mathrm{psf}} = 3$ PSF alignments, such that in this case $\varvec{\varDelta \tau } \in {\mathbb {R}}^{N_{\mathrm{psf}} M N_x N_z}$. For this case, the imaging field-of-view where PSF alignment can be effectively applied is smaller than the full field-of-view, due to limited aperture of the transducer. For instance, in Fig. 3a where $\psi _{\mathrm{psf}}=0^\circ$ is illustrated, the regions on the further right cannot be imaged, because the corresponding Rx apertures fall outside the physical aperture of the transducer.

For the PW transmissions, the relative delays from an angle separation of $\varDelta \phi$ (see Fig. 3b) are used in the SoS reconstruction. Nevertheless, the actual displacement estimations are performed using cross-correlation between PW angles with a smaller increment $\varDelta \theta$, in order to prevent speckle decorrelation and artefactual readings due to phase wrapping. To obtain the relative delay data for larger disparities $\varDelta \phi$, the delay readings from the $\varDelta \theta$ increments are then accumulated. In the literature on pulse-echo SoS imaging, delay accumulations were performed for increments of $\varDelta \theta =0.5^\circ$ as in [14, 32] or $\varDelta \theta = 2^\circ$ as in [34]. We study and compare both these settings in our experiments later below. As the choice of $\varDelta \phi$ highly affects final SoS reconstructions, we vary this parameter to find an optimal setting, as later presented in our results section.

For the DW case, the relative delays are obtained by a frame selection as illustrated in Fig. 3c. Here, the delays are directly estimated using cross-correlation-based displacement tracking between consecutive single element transmissions separated by $\varDelta \mathrm{channel}$. As can be expected, this setting highly affects the quality of the relative delays measurements: On the one hand, for a small element separation, e.g. using consecutive channels, apparent displacements can be below the tracking noise level, as also illustrated later in our experiments. On the other hand, for large element separations, coherent speckle pattern changes drastically, precluding displacement estimation. Given this tradeoff, an optimal element separation $\varDelta \mathrm{channel}$ is expected, which is studied later below in the experiments.

*Speed-of-Sound Reconstruction.* Derivation of SoS maps is based on relative delay data (cf. Fig. 2) and an inverse problem formulated to reconstruct the slowness $\varvec{\hat{\sigma }}\in {\mathbb {R}}^{N_x'N_z'}$ on a $N_x'\times N_z'$ spatial grid, which is just the inverse of the SoS:

1 $$\begin{aligned} \varvec{\hat{\sigma }} = \arg \min _{\varvec{\sigma }} \Vert \mathbf{L} (\varvec{\sigma -\sigma _0}) - \varvec{\varDelta \tau } \Vert _1 + \lambda \Vert \mathbf{D} \varvec{\sigma } \Vert _1 \end{aligned}$$

The differential path matrix $\mathbf{L} \in {\mathbb {R}}^{M N_x N_z\times N_x' N_z'}$ here links the relative slowness distribution $\varvec{\sigma -\sigma _0}$ to the relative delay measurements; for instance, in Fig. 3d the delay measurement at pixel (*x*, *z*) is sensitive to SoS variation along the illustrated paths between the beamformed images *j* and *i*. The $\varvec{\sigma _0}$ describes the initial slowness, which was used to compute the delays of the beamformed RF data.

**Fig. 4:**
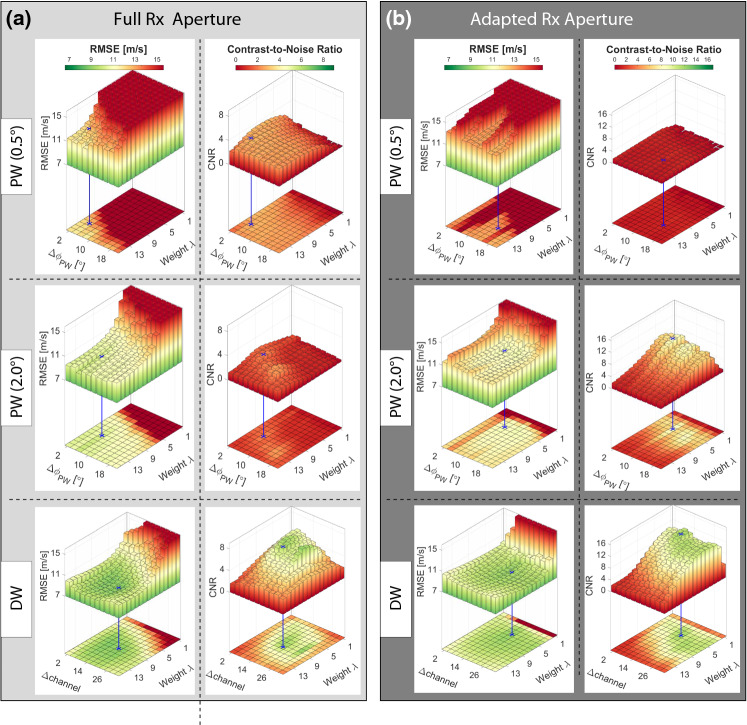
RMSE and CNR vs regularization weight, $\varDelta \phi _{PW}$ for PW, and $\varDelta \mathrm{channel}$ for DW. **a** Evaluation using full Rx aperture [14, 31], and **b** adapted Rx aperture [24, 34]. Blue bars indicate optimal parameter values for each of the 6 approaches

**Table 1:** Optimal parameter settings considering $\mathrm{RMSE}$ and $\mathrm{CNR}$ rankings, and the corresponding results of each method with best one marked in bold

Settings	Full Rx Aperture			Adapted Rx Aperture		
	PW (0.5$^\circ$)	PW (2.0$^\circ$)	DW	PW (0.5$^\circ$)	PW (2.0$^\circ$)	DW
$\varDelta$ [$^\circ$/ch]	4$^\circ$	6$^\circ$	17 ch	14$^\circ$	8$^\circ$	17 ch
$\lambda$ [a.u.]	12	10	9	11	5	5
*Results*						
$\mathrm{RMSE}$ [m/s]	12.0	9.4	**7.7**	13.12	10.0	**8.0**
$\mathrm{CNR}$ [a.u.]	$3.4\pm 4.1$	$2.7\pm 3.8$	$\mathbf{7.5\pm 10.6}$	$1.8\pm 2.3$	$10.3 \pm 9.9$	$\mathbf{14.7}\pm 11.1$

**Fig. 5:**
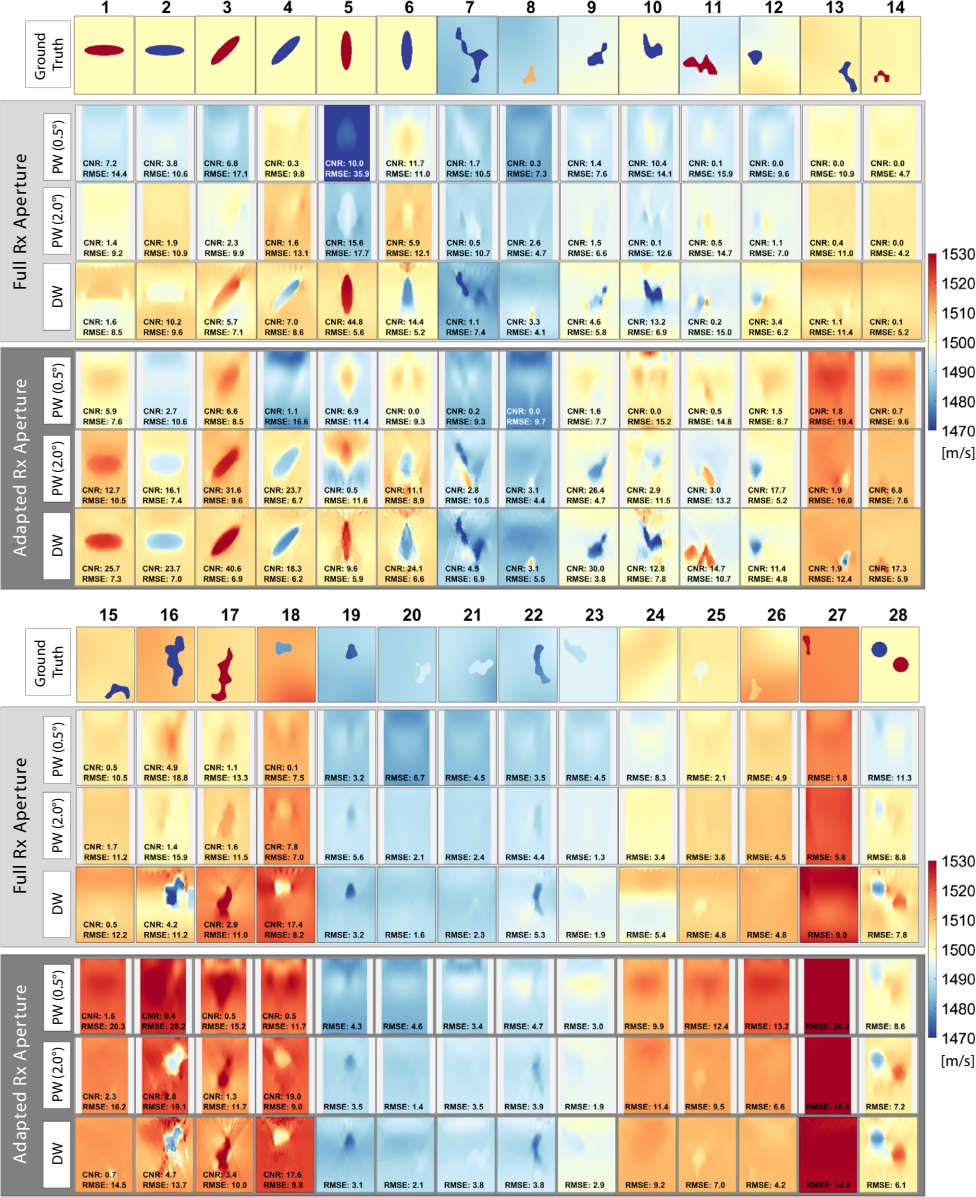
Reconstructions of 28 numerical phantoms with the optimal parametrization identified as in Fig. 4 and listed in Table 1 for PW and DW. Image dimensions are $38\,\mathrm{mm}\times 50\,\mathrm{mm}$. For PW, a small 10% margin is masked out on both sides, since the angled PW apodization cause major artifacts on the edges. Note that CNR is only evaluated for cases 1-18 having ground truth contrast of 1% or more

The regularization matrix **D** together with the weight $\lambda$ controls the amount of spatial smoothness and is essential due to ill-conditioning of $\mathbf{L}$. **D** implements LA-CT specific image filtering aimed to suppress streaking artifacts along wave propagation directions via anisotropic weighting of horizontal, vertical and diagonal gradients. For the corresponding directions either a Sobel (horizontal and vertical) or a Roberts kernel (diagonal) is used. Similarly to [31], we herein utilize a $\kappa =0.9$ anisotropic weighting. The optimization problem is solved using a limited-memory Broyden–Fletcher–Goldfarb–Shanno (L-BFGS) algorithm [4, 6, 9, 33].

**Fig. 6:**
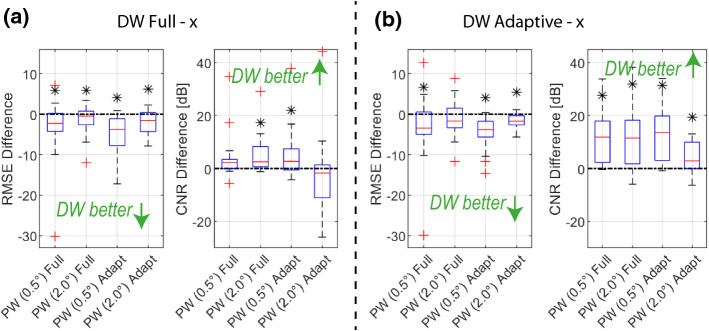
Improvements in RMSE and CNR for (a) DW Full Aperture and (b) DW Adaptive Aperture, compared to each PW method in a paired fashion. Stars indicate significant improvements with $p<0.05$

**Fig. 7:**
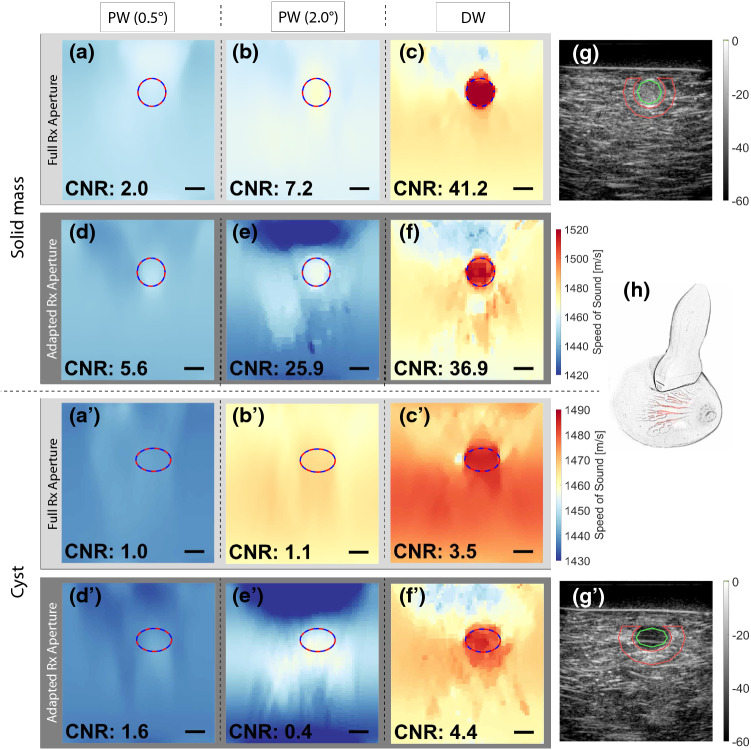
DW and PW based SoS reconstructions for two different cross sections of the CIRS breast phantom (Multi-static raw RF data and DW reconstruction results, together with inclusion masks and an evaluation routine are provided as supplementary material)  (**a**–**f**, **a**’–**f**’) with the lesion delineations derived from B-Mode images (**g**, **g**’). Green/red contours represent the regions inside/outside the lesions used for $\mathrm{CNR}$ analysis. (**h**) Sketch of the imaging setup with the linear array probe on the breast phantom. Scalebars: $5\,\mathrm{mm}$

For computational efficiency, we restrict the number of relative delay data readings $\varvec{\varDelta \tau }$ in eq. (1) to $10^4$, which are randomly selected from all ${\mathbb {R}}^{M N_x N_z}$ (full Rx aperture) or ${\mathbb {R}}^{N_{\mathrm{psf}} M N_x N_z}$ (adapted Rx aperture) recordings, respectively.

## Materials and experiments

*Numerical simulations.* To evaluate how accurate SoS heterogeneities can be imaged using the above explained SoS imaging method based on PWs or DWs, we simulated a pulse-echo scenario, where a linear transducer is simulated and the echos at each element are recorded. In total 28 SoS heterogeneity cases are simulated (see first rows in Fig.5a/b), which are divided into two subsets.

The first subset (cases 1-6 and 28) consists of seven defined shapes on a homogeneous background substrate of $1500\,\mathrm{m}/\mathrm{s}$. Elliptical and circular inclusions have a SoS contrast of either $-2\%$ (i.e. $1470\,\mathrm{m}/\mathrm{s}$) or $+2\%$ (i.e. $1530\,\mathrm{m}/\mathrm{s}$). The last case has two circular inclusions. The second subset (7–27) consists of randomly shaped inclusions (SoS values: [$1450,1550]\,\mathrm{m}/\mathrm{s}$). Substrate SoS values are varied in two ways: (1) Average SoS of the substrate is no longer fixed to $1500\,\mathrm{m}/\mathrm{s}$, but take values between [$1485,1515]\,\mathrm{m}/\mathrm{s}$. (2) Each substrate is varied locally between $\pm 3\,\mathrm{m}/\mathrm{s}$. Such substrate variations are important to evaluate how reconstructions would perform with natural tissue variation. To allow for displacement estimation (cf. Fig. 2), a fully-developed speckle pattern is required, realized herein by increasing a random 10% set of the medium pixels by a slight density perturbation.

A linear array transducer is modeled with $N_c = 128$ channels and a $300\,\mu \mathrm{m}$ pitch. We used transmit pulses of $f_c=5\,\mathrm{MHz}$ center frequency with 3 half cycles. All simulations (including in Fig. 1) were run with a spatial discretization of $75\mu \hbox {m}$ pixels and a temporal resolution of $6.25\,\mathrm{ns}$ (i.e., $160\,\mathrm{MHz}$ sampling frequency) to allow for an accurate sampling of the wave propagation. Herein, for each case a full-matrix capture with multi-static transmission was simulated first and then recomposed into corresponding PWs or DWs using synthetic aperture Tx/Rx beamforming. To reduce high frequency artifacts, we additionally applied a 60% band-pass filter on the recomposed RF data.

For experiments, 81 PW angles (ranging between $-20^\circ$ and $20^\circ$ with a step size of $0.5^\circ$ and Tukey apodization) were simulated via synthetic-aperture. For DW, we used single element multi-static transmissions (see Fig. 3a/b). All raw data was beamformed based on a constant SoS assumption of $1500\mathrm{m}/\mathrm{s}$.

*Tissue-mimicking phantom.* For data acquisition of the breast phantom (CIRS Multi-Modality Breast Biopsy and Sonographic Trainer, Model 073, CIRS Inc., Norfolk, VA, USA), unbeamformed RF data using a UF-760AG ultrasound system (Fukuda Denshi, Tokyo, Japan) were recorded with a FUT-LA385-12P linear array transducer ($N_c = 128$ channels, $300\,\mu \mathrm{m}$ pitch and 4 half cycles pulses of $f_c=5\,\mathrm{MHz}$ center frequency. To increase the signal-to-noise ratio, data was transmitted using Walsh-Hadamard coded pulses [38] with subsequent recomposition into angled PWs or DWs, respectively. After recomposition, a 60% band-pass filter was applied. Beamforming was performed based on a constant SoS assumption of $1470\mathrm{m}/\mathrm{s}$, which is the approximate nominal SoS value of the phantom substrate.

*Evaluation metrics.* For a quantitative analysis of the SoS reconstruction $\hat{\varvec{c}} =1/\hat{\varvec{\sigma }}$ in simulation, we used Root-mean-squared-error ($\mathrm{RMSE} =\sqrt{\Vert \hat{\varvec{c}} -\varvec{c}^\star \Vert _2^2 / N}$) and Contrast-to-noise ratio ($\mathrm{CNR} = 2(\mu _{\mathrm{inc}}-\mu _{\mathrm{bkg}})^2/(\sigma _{\mathrm{inc}}^2 + \sigma _{\mathrm{bkg}}^2)$), given mean $\mu$ and variance $\sigma ^2$ of $(\cdot )_{\mathrm{inc}}$ and $(\cdot )_{\mathrm{bkg}}$, respectively denoting the region of the inclusion and the background. In the simulation datasets, the $\mathrm{CNR}$ was only computed for cases 1-18, where the inclusion had an SoS contrast is at least 15 m/s, i.e. 1$\%$ compared to the substrate. The background values were computed based on the whole substrate region for the simulated datasets.

## Results and discussion

*Simulation study.* First, a sensitivity analysis with respect to major parametrization choices was performed for the corresponding transmission sequences (PW and DW). We used a simulated phantom dataset of 28 ground-truth SoS distributions, representative of different characteristics in inclusion shape, size and SoS contrast as well as background SoS variations. These datasets are then evaluated in terms of $\mathrm{RMSE}$ as well as in terms of $\mathrm{CNR}$, indicating how well the inclusions can be separated from the background, which is of major importance in the context tumor detection/characterization. The optimal parameters ($\varDelta \phi$, $\varDelta \mathrm{channel}$ and regularization weight $\lambda$) are then selected as follows: For each parameter combination, average $\mathrm{RMSE}$ and $\mathrm{CNR}$ across all sample images was computed, as also plotted in Fig. 4. These values were then ranked from best to worst (i.e., ascending order for RMSE, and descending for CNR), and the optimal parameter set (cf. Table  1) was chosen as the one minimizing the average rank of the two metrics. The results are also summarized in Table 1, where it can be seen that with DW the best results are achieved with an overall $\mathrm{RMSE}=7.7$ and 8.0, respectively, for full and adapted Rx aperture cases. For the PW case, the best achievable results are at least 1.7m/s on average poorer with $\mathrm{RMSE}=9.4$ and 10, respectively. The contrast is also substantially improved with DW to $\mathrm{CNR}=7.5$ and 14.7, respectively, with over 42% improvement compared to best case PW results of $\mathrm{CNR}=3.4$ and 10.3.

Using the determined optimal parameter settings, the reconstructions of the 28 test images are shown in Fig. 5. The DW-based SoS reconstructions are seen to be superior to PW-based in almost all cases. Especially with the case 28 in Fig. 5 with both higher and lower inclusions, DW is seen to perform significantly superior, regardless of the choice of aperture. The adapted RX aperture is especially beneficial with DW in layered structures such as shown in cases 1 and 2. This may be thanks to the smaller RX aperture leading to a higher coherence in delayed signal summation when beamforming, thus improving displacement tracking and hence SoS reconstruction. Some reconstruction errors are seen when the inclusion is very deep (e.g., simulations 15 and 26), when there cannot be sufficiently many lag measurements within the field-of-view below an inclusion, to help drive its reconstruction. Similarly, when an inclusion is located to the sides (e.g., simulation 27), many Tx apertures may not cover it, again reducing lag measurements for its reconstruction. Accordingly, where possible, an inclusion should be imaged in the middle of the imaging field for optimal reconstructions [27, 31]. For the PW cases, the best reconstructions are achieved using an angle accumulation of $\varDelta \theta =2^\circ$ and an adapted receive aperture, similarly to [34]. It is worthwhile to note that the adaptive receive aperture setting with a similar $\mathrm{RMSE}$ (of 10.0 vs. 9.4 m/s) compared to the full receive aperture setting, leads to substantial improvement in $\mathrm{CNR}$ (of 10.3 vs. 2.7). A similar trend is observed in the DW case with adapted vs. full receive aperture settings ($\mathrm{RMSE}$: 8.0 vs. 7.7 m/s; $\mathrm{CNR}$: 14.7 vs. 7.5). Notwithstanding the aperture differences, the overall SoS imaging is significantly improved using DW vs. PW.

Improvements in average metrics is corroborated using a paired hypothesis test as shown in Fig. 6, where the DW methods (using either a full or adaptive aperture) are compared for each sample against all other PW methods. Using a full receive aperture setting in DW results in significant RMSE improvement compared to any other PW method, irrespective of the PW receive aperture setting. Furthermore, significant CNR improvement w.r.t. any PW method is indicated using DW with adaptive aperture.

Note that for PW we focus on the center part of the image and mask out 10% on both sides of the imaging region (Fig. 5), since the apodization of the angled PW causes significant artifacts in these image regions, as was also discussed in [27]. Accordingly, $\mathrm{RMSE}$ and $\mathrm{CNR}$ were computed in these shown central regions. For a fair comparison, this same region is also used for computing the DW metrics, even though this is not a limitation in DW imaging and such masking is not required as depicted in Fig. 5.

Beamforming was conducted assuming a constant $1500\,\mathrm{m}/\mathrm{s}$, although the actual background SoS differed sometimes over $15\,\mathrm{m}/\mathrm{s}$. Despite such deviations between actual and beamforming SoS, the reconstructions are seen to still perform relatively well, as can be seen, e.g. in case 8 with an inclusion as well as in cases 20 and 25 with nearly homogeneous SoS distributions. Indeed, these examples indicate that it is possible to use our estimated SoS values in the beamforming process, as shown in [24], and potentially extend this to further refine SoS reconstructions iteratively. This is relevant to real-case scenarios where the exact SoS values are not known a priori.

*Phantom experiment.* CIRS breast phantom has stiff inclusions representing malignant solid masses with higher speed-of-sound (cf. Fig. 7g), and hypoechoic inclusions representing cysts (cf. Fig. 7g’), which have smaller SoS contrast with its surrounding. We reconstructed SoS maps using the optimal settings found in the previous section (cf. Table 1), since we modeled this probe and acquisition scheme in our simulations. to the wide range of experimental settings studied herein (i.e. varying contrast, inclusion size, inclusion shape, background SoS for both, simulated and phantom data), the derived optimal settings are hoped to generalize to a variety of applications and tissue types. For different imaging device and probe characteristics, these may however need to be reparametrized. SoS reconstructions using the different studied methods are shown in Fig. 7a-f and a’-f’. Similar to a few cases in the simulation study, different methods can result in overall different absolute SoS offset, which is believed to be caused by strong aberration effects and corresponding inaccurate shifts delay estimations. The artifactual SoS offsets were represented in the simulation study by the $\mathrm{RMSE}$, which was seen to be superior in the DW cases. Hence it can be assumed that the DW methods in the phantom study also result in more accurate absolute SoS estimations. Furthermore the DW approach is seen to substantially improve the detection of both the solid mass and the cyst, whereas with PW neither the inclusion shows contrast nor the background SoS appears consistent. This is also reflected by the $\mathrm{CNR}$ improvement as shown in the corresponding figure corners. For the solid mass, which has a high SoS contrast, the DW approach leads to a $\mathrm{CNR}$ improvement of more than 42%. For the more challenging case of the cyst-representative inclusion with lower SoS contrast, the improvement is even more substantial with a 2.8-fold better $\mathrm{CNR}$.

Regarding the optimal choice of Rx aperture for the breast phantom datasets, it was observed that for PW adapted Rx apertures often lead to significantly improved results (except for the 2$^\circ$ PW of cyst inclusion where the CNR is generally very low due to low contrast nature of this inclusion). For DW, however, adapted Rx apertures only yields a marginal improvement in $\mathrm{CNR}$ for the cyst case, while being inferior for the high contrast inclusion. The reason for this might be that for the full Rx aperture case, a higher regularization weight ($\lambda _{\mathrm{full }}=9$ vs. $\lambda _{\mathrm{adap}}=5$) was found to be optimal in our parametrization study. This leads to higher spatial smoothness and a reduced noise in the background, thus potentially boosting the $\mathrm{CNR}$ of high contrast masses. A high regularization would negatively affect the detectability of low contrast inclusions, as these are more prone to be smoothed out during reconstruction, which corroborates the observation in Fig. 7c’.

We herein compared DW and PW sequences that would theoretically yield a similar framerate, i.e. with the setting $M=9$ a total of 18 transmit events needed with either method for one SoS frame (neglecting any compounding preprocessing or SNR-boosting steps that may be used for either method). With DW, although each Tx yields less measurements for reconstruction, due to limited aperture, this in turn speeds up computations for beamforming, time-delay measurements, and subsequent optimization. A major drawback of DW with a single element on a physical system would be the limited Tx energy and hence a low SNR. For the experimental example, we address this herein with Walsh-Hadamard coded pulses [38], which excite the tissue with sufficient power while they can be mapped linearly to any single element combination (DW Tx event) retrospectively. Since such a coded imaging approach may reduce frame-rates for in-vivo applications, an alternative way of inducing DWs with high SNR and without loss of frame-rate would be to utilize virtual source transmit, where a DW is formed using multiple transducer elements.

## Conclusion

We have presented herein the use of diverging waves (DW) in pulse-echo SoS image reconstruction, studying it comparatively to existing plane waves (PW) approaches. Analyzing the wavefront aberrations with PW and DW insonifications, DW was seen to cause less aberration artifacts that lead to inaccuracies in displacement estimation. This improved delay estimation applies irrespective of chosen linear-path forward model and ray discretization assumptions for SoS reconstruction. Motivated by this, we have studied a set of numerical phantoms, observing that the quantitative accuracy ($\mathrm{RMSE}$) of SoS reconstructions is over 20% improved by using DW compared to PW. Even more pronounced are the improvements in inclusion contrasts, where $\mathrm{CNR}$ led to an improvement of over 42% with DW. These results are corroborated by an actual ultrasound acquisition of a breast phantom, where $\mathrm{CNR}$ improvements of more than 42% and 280% are achieved with DW for, respectively, high and low contrast inclusions.

Diverging waves in this work are generated without loss of generality using a single element transmission yielding circular wavefronts. Nevertheless, the presented method after minor adjustments of *L* matrix paths and beamforming delays is also applicable for multiple-element transmission using a virtual source approach and also to non-circular wavefronts, which would allow to increase the echo SNR. With our findings SoS imaging based on conventional ultrasound systems can be substantially improved, paving the way for translating SoS imaging into the clinic. Quantitative SoS imaging and its improvements as presented herein are not only valuable in diagnostic and interventional imaging, but would also help improve many other ultrasound-based modalities by correcting aberrations, such as improved beamforming for higher-quality B-mode images as presented in [24].
